# Radiofrequency Ablation-Assisted Zero-Ischemia Robotic Laparoscopic Partial Nephrectomy: Oncologic and Functional Outcomes in 49 Patients

**DOI:** 10.1155/2016/8045210

**Published:** 2016-12-01

**Authors:** Kalen Rimar, Aziz Khambati, Barry B. McGuire, David A. Rebuck, Kent T. Perry, Robert B. Nadler

**Affiliations:** Department of Urology, Northwestern University Feinberg School of Medicine, Chicago, IL, USA

## Abstract

*Introduction and Objectives.* Robotic partial nephrectomy with peritumoral radiofrequency ablation (RFA-RPN) is a novel clampless technique. We describe oncologic and functional outcomes in a prospective cohort.* Methods*. From May, 2007, to December, 2009, 49 consecutive patients with renal masses <7 cm underwent RFA-RPN. During this period, only the RFA-RPN technique was utilized for all cases of partial nephrectomy. Pre- and postoperative data were analyzed and compared to 36 consecutive patients who underwent LPN.* Results*. In total, 49 tumors were treated in the RFA-RPN group and 36 tumors in the comparison group. Mean operative time was longer in the RFA-RPN group (370 min versus 293 min, *p* < 0.001). There were no significant differences in mean EBL (231 cc versus 250 cc, *p* = 0.42), transfusion rate (8.2% versus 11.1%, *p* = 0.7), or hospital stay (3.9 versus 4.4 days, *p* = 0.2). Two patients in the RFA-RPN (4.1%) and 1 (2.7%) patient in the comparison group had a positive surgical margin (*p* = 0.75). 17 (34.7%) patients had a postoperative urine leak in the RFA-RPN group versus 2 (5.6%) patients in the comparison group (*p* = 0.001). Mean follow-up was 54 months versus 68.4 months in the comparison group. There was no significant difference between the two groups regarding change in GFR (*p* = 0.67). There were 3 recurrences (6.1%) in the RFA-RPN group and 0 recurrences in the RPN group (*p* = 0.23). There were 3 deaths (6.1%) in the RFA-RPN group (one cancer specific) and 4 deaths (11.1%) in the RPN group (non-cancer specific) over the follow-up period (*p* = 0.44).* Conclusions*. Our data suggests that this technique is associated with a similar degree of renal preservation but higher rates of postoperative urine leak and possibly higher rates of recurrence.

## 1. Introduction

The incidence of renal cell carcinoma is increasing [1, 2] due to increased cross-sectional imaging, resulting in a stage shift to tumors less than 4 cm (small renal masses (SRM)) [3]. Management strategies for the SRM include radical/partial nephrectomy, tumor enucleation, thermal ablative therapies, or surveillance. Although EORTC 30904, the only randomized controlled trial of radical nephrectomy versus partial nephrectomy, did not show any survival benefit with nephron sparing surgery at a median follow-up of 12 years, partial nephrectomy remains the treatment of choice for SRM. The reasons for this are many: approximately 30% of SRMs that will be benign [3], the risk of chronic kidney disease and/or end stage renal disease which may result in dialysis, and the risk of developing a contralateral tumor. As such, efforts to find a surgical solution that is minimally invasive and of low risk and confers reduced morbidity are continuously sought after.

Ablative strategies, such as cryoablation [4, 5] and radiofrequency ablation (RFA) [6], are used to treat SRMs, and although they are considered safe with early acceptable oncological control, long term data on their efficacy is needed [6]. We previously described a novel technique of radiofrequency ablation-assisted robotic clampless partial nephrectomy (RFA-RPN) [7, 8], a technique in which RFA energy is used to create a coagulated plane around the tumor, and the tumor is excised with zero ischemia. The aim of this technique was to allow excision of the tumor with reduced bleeding without clamping the renal vessels and thereby preventing injury through warm ischemia. In this initial study, there were no differences in blood loss, transfusion, and complication rates when compared to standard robotic partial nephrectomy in the perioperative period [8]. We now, in a larger series, report postoperative, functional, and oncologic outcomes at a mean follow-up of 54 months in patients who underwent RFA-RPN and 68.4 months in the standard robotic partial nephrectomy (RPN) comparison group.

## 2. Materials and Methods

### 2.1. RFA-RPN Technique

From May, 2007, to December, 2009, 49 consecutive patients underwent RFA-RPN. During this period, only the RFA-RPN technique was utilized for all cases of laparoscopic partial nephrectomy. Our RFA-RPN technique has been described in detail [7, 8]. In short, a 7Fr ureteral catheter was inserted at the start of the case to allow for retrograde injection of methylene blue for intraoperative assessment of entry into the collecting system. The kidney and renal hilum were dissected using a transperitoneal pure laparoscopic approach utilizing the robotic ports. Laparoscopic ultrasonography was then performed to locate the tumor. The da Vinci robot (Intuitive Surgical, Sunnyvale, CA) is then docked. The Habib 4X bipolar RFA device (AngioDynamics, Queensbury, NY) coupled to the Rita 1500X (software version 8.41) generator was then used to create a 0.5 cm plane of coagulated tissue at the interface between the tumor and normal parenchyma. The endpoint of RFA application was impedance and repeat applications were performed until reaching the endpoint that could be reached in ≤ 5 seconds, as measured by the audible beep on the generator [7]. While the RFA device itself does not articulate, with full renal dissection, the surgeon at the robotic console was able to position the kidney to allow the bedside assistant-controlled RFA device to use the best angle of approach. The robotic cold scissors were then used to excise the tumor. The RFA device was used to control any bleeding as needed. The duration of RFA energy application was recorded. The methods for repair of the collecting system were similar in both groups. If injury to the collecting system was identified intraoperatively, it was repaired with running 4-0 absorbable suture. In the RFA-RPN group, the repair was gently tested with injection of methylene blue to ensure a water tight seal. Surgical bolsters and FloSeal were then placed in the tumor bed, followed by a sliding-clip renorrhaphy. Once frozen section of tumor bed biopsies and margins were confirmed negative for residual tumor, a closed suction drain was placed, and we proceeded with closure.

### 2.2. Patient, Tumor Characteristics, Perioperative Outcomes, and Complications

Patient and tumor characteristics were recorded. Endophytic tumors were defined as those with >50% of tumor volume within normal kidney outline or tumor impinging upon the collecting system as seen on preoperative imaging [9]. We also calculated the RENAL nephrometry score [10]. A postoperative urine leak was defined as the presence of any urine in the drain (drain fluid creatinine greater than serum). The duration and management of the urine leak were recorded. The Clavien-Dindo classification of surgical complications was used [11]. We considered a postoperative urine leak not requiring any intervention (other than continued drainage) to be a grade-2 complication. A delayed hemorrhagic complication was defined as any evidence of bleeding occurring after discharge from the hospital.

### 2.3. Comparison Group

From October 2002 to May 2007, 36 consecutive patients underwent RPN with renal hilar vessel clamping and cold sharp excision of renal tumors as previously described [7]. Starting from June 2007, only the RFA-RPN technique was utilized for partial nephrectomy until November 2009.

### 2.4. Functional and Oncologic Outcomes

Patients were followed up radiographically with CT (or MRI) and chest X-ray at 6 months and then yearly thereafter. The patients' renal function was also assessed at each clinic visit. Renal scans were not routinely performed unless clinically indicated. The duration of follow-up was based on the date of the most recent abdominal imaging. We also recorded whether any patients required nephrectomy (for disease recurrence, recurrent infection, fistula, or pain). The study was approved by Northwestern Institutional Review Board approval (study number STU00012456). SPSS™ (IBM, Illinois, USA) was used to perform the statistical analyses.

## 3. Results

### 3.1. Perioperative Outcomes

In total, 49 tumors were treated in 49 patients.  Table 1 summarizes the patient and tumor characteristics for both the RFA-RPN and RPN comparison group.  Table 2 summarizes the perioperative outcomes. All RFA-RPN procedures were zero ischemia (clampless) and there were no conversions. The mean tumor size was 2.6 cm and 28.6% of all tumors were ≥ 3 cm compared to 2.0 cm and 8.3% of tumors in the RPN group (*p* = 0.004 and 0.016, resp.). The mean RENAL nephrometry score was 5.7 (range: 4–12). 57.1% of tumors were considered endophytic compared to 16.1% in the RPN group (*p* < 0.001). Mean operative time was 370 minutes in the RFA-RPN group versus 293 minutes in the comparison group (*p* < 0.001). The mean duration that the RFA device was activated was 25.5 minutes. There were no significant differences in estimated blood loss (EBL) (231 mL versus 250 mL, *p* = 0.42), rate of transfusion (8.2% versus 11%, *p* = 0.7), rate of nonurologic complications (10.2% versus 13.9%, *p* = 0.7), rate of malignant tumor (69.4% versus 66.7%, *p* = 0.8), or mean hospital stay (3.9 days versus 4.4 days, *p* = 0.2).

In the RFA-RPN group, 2 patients (4.1%) had a positive surgical margin, as 1 patient (3.3%) in the RPN group (*p* = 0.75). 17 (34.5%) patients in the RFA-RPN group had a postoperative urine leak compared to 2 (5.6%) patients in the RPN group (*p* = 0.001). The majority (15 cases) were successfully managed with prolonged closed suction drainage with or without ureteral stenting with drain removal after a mean of 7.5 days (range: 3–19). The rate of subsequent procedures/admissions required was 16.3% in the RFA-RPN group versus 11.1% in the RPN group (*p* = 0.57). When comparing patients with and without urine leaks, the urine leak group had a higher proportion of patients with ASA score ≥ 3 (35.3 versus 18.8%, *p* < 0.001), a higher incidence of collecting system repair during renorrhaphy (94.1 versus 65.6%, *p* = 0.021), and a trend towards a higher mean RFA duration (30.7 versus 22.3 minutes, *p* = 0.087).

Due to falling hemoglobin in the first eight hours postoperatively, early laparoscopic reoperation occurred in 2 (4.1%) patients, revealing venous bleeding in the RFA-RPN group. Areas were oversewn with absorbable suture in a figure of eight fashions. There were no cases of delayed hemorrhage in either group.

### 3.2. Functional and Oncologic Outcomes

The mean follow-up was 54 months (range: 2.4–84) in the RFA-RPN group, and 46 patients (91.8%) had follow-up of at least 6 months compared to 68.4 months (range: 1.0–129.6) in the RPN comparison group, with 33 patients (91.7%) having follow-up of at least 6 months (*p* = 0.53). The mean decrease in GFR for the RFA-RPN group was −14.8 mL/min/1.73 m^2^ (range +16–−56, SD 17.4, 95% CI 5.4) versus −16.5 mL/min/1.73 m^2^ (range +24–−50, SD 16.7, 95% CI 6.2) (*p* = 0.67) in the RPN comparison group. At the time of the most recent follow-up visit, no patients in either group were on renal replacement therapy. One patient (2%) in the RFA-RPN group developed symptomatic ureteropelvic junction obstruction with poor ipsilateral function on diuretic renal scan and required simple nephrectomy.

In the RFA-RPN group, there were 3 (6.1%) disease recurrences; 2 local recurrences and 1 metastatic recurrence. There were no recurrences in the RPN comparison group (*p* = 0.23). Both local recurrences occurred two years after the initial procedure (T1a grades 1 and 2, resp.) and required completion radical nephrectomy. The patient with a Fuhrman grade 2 tumor also had a contralateral, enhancing 1.9 cm lesion noted at the time of recurrence and chose to undergo cryoablation of the contralateral mass concurrently with completion radical nephrectomy. This patient was discovered to have brain metastasis 1 year later after presenting with neurologic symptoms. The third patient had metastatic disease at 13 months and expired after progression of his disease on systemic chemotherapy 23 months after the date of surgery. All patients with disease recurrence had negative surgical margins at the time of their initial surgery. There were 3 total deaths (6.1%) in the RFA-RPN group over the follow-up period including the 1 cancer specific death described previously. The remaining 2 patients died of a myocardial infarction 60 months after the date of surgery, and renal failure 12 months after the date of surgery. There were 4 deaths (11.1%, *p* = 0.44) in the RPN comparison group, none of which were cancer specific. Two patients died from heart failure exacerbations >5 years from the date of surgery. One patient died of metastatic squamous cell carcinoma (nonrenal primary, renal pathology papillary RCC) >5 years from the date of surgery. One patient died from cholangiocarcinoma which was incidentally discovered on imaging for his renal cell carcinoma follow-up 30 months after the original date of surgery.  Table 3 summarizes the functional and oncologic outcomes.

**Figure 1:**
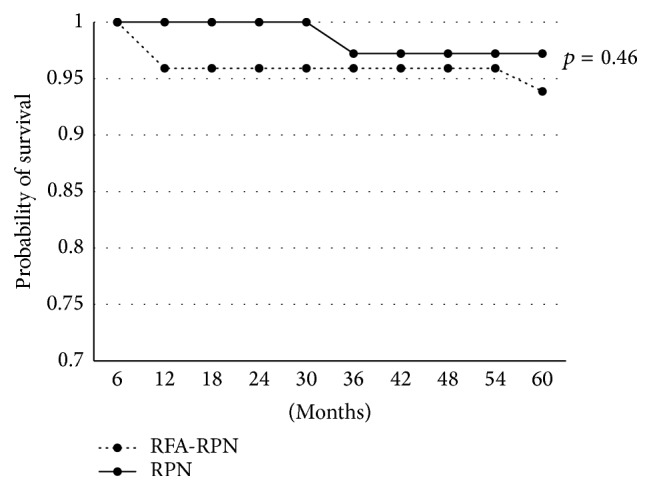
Comparison of overall survival. Five-year overall survivals were compared between the RFA-RPN and RPN groups.

**Figure 2:**
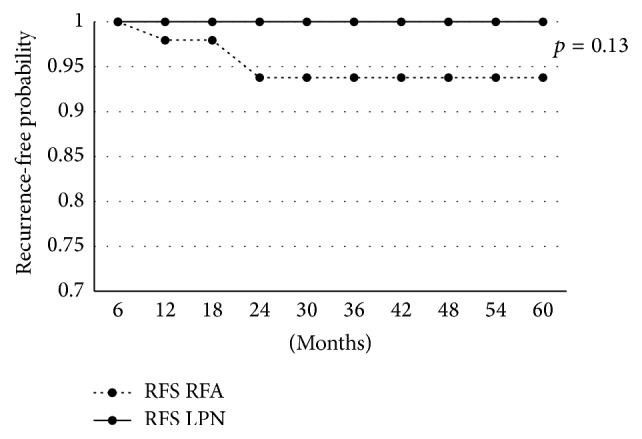
Comparison of recurrence free survival. Five-year recurrence-free survivals were compared between the RFA-RPN and RPN groups.

The five-year overall survival for the RFA-RPN group was 93.8% versus 97.2% in the RPN group (*p* = 0.46) (see Figure 1). Five-year recurrence-free survival for the RFA-RPN group was 93.7% versus 100% for the RPN group (*p* = 0.13) (see Figure 2). Five-year cancer specific survival for the RFA-RPN group was 97.9% versus 100% for the RPN group (*p* = 0.37).

## 4. Discussion

We describe the postoperative, renal and oncological outcomes in 49 patients who underwent a novel technique of radiofrequency ablation-assisted robotic partial nephrectomy (RFA-RPN) with zero ischemia. By using RFA energy to create a plane of coagulated tissue around the tumor, our technique attempts to reduce hemorrhage and eliminate warm ischemia associated with LPN. We also compared this data to our consecutive series of 36 patients who underwent standard RPN with renal hilar vessel clamping and cold sharp excision of renal tumors from October 2002 to May 2007. Our data shows that renal function is reasonably maintained utilizing our technique; however, it is associated with a significantly higher rate of postoperative urine leak when compared to the standard RPN. In addition, one patient required nephrectomy from a UPJ obstruction presumably caused by thermal injury. While there have been several other studies that have reported their experience with RFA-assisted LPN [9, 12–20], this series is unique for several reasons: First, robotic assistance was utilized; second, RFA energy was not used to ablate the tumor itself but to coagulate a plane between tumor and normal parenchyma of the kidney in a clampless partial nephrectomy; and, third, our cohort has the longest follow-up of all RFA-assisted RPN series reported to date.

The most striking finding in our cohort was the high rate of postoperative urine leak of almost 35% in the RFA-RPN group. This rate is significantly higher than others reported in the literature and was significantly higher than the rate in our comparison group (5.6%). Important differences in patient characteristics as well as the use of RFA in combination with tumor excision may account for this. Previous studies of monopolar RFA-assisted LPN in the porcine model demonstrated that radiofrequency energy does not seal collecting system violations [21]. This may also apply to bipolar RFA, as what was used in this cohort. Furthermore, a zone of parenchymal necrosis ranging from 3 to 9.6 mm may be induced around the active tine [19, 20, 22]. RFA is typically used to ablate the tumor itself. However, in our study, the RF energy was used adjacent to the tumor to allow for resection without clamping of the renal hilum. Thermal injury from prolonged RFA time may result in delayed tissue and/or repair breakdown that is not apparent to the surgeon at the time of closure. Tumor resection after peritumoral ablation, rather than ablation of the tumor itself, may explain our high leak rates as the zone of parenchymal necrosis may be more likely to involve the collecting system repair. Tumor complexity may have also played a role as 57% of tumors were endophytic and, in almost 28.6% of cases, the tumor size was ≥ 3 cm. Such tumors tend to be more centrally located, can be more difficult to resect, and are more likely to involve the collecting system. Patients with urine leaks were also more likely to have a higher ASA score, indicating that patients with poorer overall health status were at a greater risk for postoperative urine leak. While the relationship between leak status and RFA duration was not statistically significant, we believe our study was likely underpowered to detect a difference. The mean duration that RFA energy used was 25.5 minutes and may be too long, considering the urine leak rate and unusual complication of UPJ obstruction, which could be assumed to be from thermal injury. The approach of peritumoral (rather than intratumoral) RFA application likely explains the increased RFA durations required for this approach, as multiple RFA needle insertion was required to surround the entire periphery of the tumor. Furthermore, despite administration of high thermal energy levels, this did not translate into lower hemorrhagic risks; the transfusion rate of 8.2% is consistent with our comparison group of robotic partial nephrectomy but slightly higher than most modern series of LPN in the literature [23–25].

A high recurrence rate (6.1%) in the RFA-RPN group was observed, though not statistically significant. All three patients had low complexity tumors based on nephrometry score (4p, 4p, and 6p), negative margins, and low risk tumors (clear cell, Fuhrman grade < 2, size <4.0 cm). Though our small sample size limits our interpretation, we suspect that the RFA energy at the tumor border could make it more difficult to determine accurate margin status particularly on frozen section. All three patients had their procedures performed >1 year into the study making it unlikely that the learning curve of the procedure played a role.

Weaknesses of our study include the lack of randomization between the two treatment groups as well as the relatively low number of overall patients. Though the surgeons performing the procedure were experienced with the robotic technique, this was within the relative infancy of widespread robotic surgery and may make comparison with modern series more difficult. This initial study raises a number of important questions regarding the complications and oncologic efficacy associated with this procedure. Larger studies with longer follow-up would likely be needed to more accurately determine the perioperative, functional, and oncologic outcomes. Due to the high urine leak rates and equivalent functional outcomes, this procedure is no longer performed at our institution.

## 5. Conclusion

In an effort to minimize patient morbidity while maintaining cancer control, ongoing evolution and adoption of new surgical techniques is essential. In the largest series of patients who underwent zero-ischemia RFA-RPN, we describe our perioperative, functional, and oncological outcomes. Renal functional outcomes are similar. However, there are a high rate of postoperative urine leak and a concerning trend regarding cancer recurrence. Further research into the thermal injury effects of this technique is required, and any potential benefits should be described in the setting of a large randomized controlled trial.

## Figures and Tables

**Table 1:** Patient and tumor characteristics.

	RPN		RF-RPN		*p*
Number of patients	36		49		
Mean age at diagnosis (range), years		58.4 (36–79)		58.5 (30–77)	0.37
Number of men (%)		22 (61%)		27 (55%)	0.66
Mean BMI^a^ (range), kg/m^2^		26.7 (19.2–41.0)		29 (20.0–55.0)	0.1
ASA^b^ score >3	34	11 (30.6%)	45	11 (24.4%)	0.46
Mean pre-op GFR^c^, mL/min/1.73 m^2^		83.9		80.7	0.48
Clinical tumor size	36		49		
Mean (range), cm		2.0 (0.7–6.0)		2.6 (0.9–6.0)	0.004
Size > 3 cm (%)		3 (8.3)		14 (28.6)	0.016
Endophytic	31	5 (16.1%)		28 (57%)	<0.001
Location (%)	31		49		0.5
Upper		13 (36.1)		20 (40.8%)	
Interpolar		17 (47.2)		17 (34.7%)	
Lower		6 (16.7)		11 (22.4%)	
Left-sided tumor		28 (77.8%)		25 (51%)	0.013
Mean follow-up with imaging, months (range)		68.4 (1.0–129.6)		54 (2.4–84)	0.053
Patients with follow-up of at least 6 months (%)		33 (91.7%)		46 (91.8%)	0.69

^a^BMI, Body Mass Index.

^b^ASA, American Society of Anesthesiologists.

^c^GFR, glomerular filtration rate.

**Table 2:** Perioperative outcomes and complication.

	RPN		RF-RPN		*p*
Mean warm ischemia time, min		31.1		0	
Mean total operative time, min		293		370	<0.001
EBL^a^					
Mean (range), cm		250 (100–800)		231 (50–1200)	0.42
Number of transfusion (%)		4 (11.1%)		4 (8.2%)	0.7
Mean hospital stay (range), days		4.4 (2–12)		3.9 (1–14)	0.2
Mean pathologic tumor size, greatest dimension (range), cm		3.7 (1.5–12)		4.1 (1.2–12)	0.31
Number of pathological diagnosis:	36		49		
Renal cell carcinoma (%)		24 (66.7%)		34 (69.4%)	0.8
Benign kidney findings (%)		12 (33.3%)		15 (30.6%)	0.8
Positive surgical margin (%)		1 (3.3%)		2 (4.1%)	0.75
Other malignancies (%)		0 (0%)		0 (0%)	
Number of cases requiring collecting system reconstruction (%)		11 (30.6)		37 (76%)	<0.001
Number of patients with urological complications (%)		3 (8.3%)		18 (36.7%)	0.004
Number of patients with urine leak (%)		2 (5.6%)		17 (34.5%)	0.001
Number of patients with nonurological complications excluding hemorrhage (%)		5 (13.9%)		5 (10.2%)	0.7
Number of patients with hemorrhage (%)		4 (11.1%)		3 (6.1%)	0.45
Subsequent procedures/admission needed (%)		4 (11.1%)		8 (16.3%)	0.57

^a^EBL, estimated blood loss.

**Table 3:** Functional and oncologic outcomes.

Outcome	RFA-RPN group	RPN group	*p* value
	Mean (range) or *n* (%)	Mean (range) or *n* (%)	
Change in renal function over follow-up (GFR^a^, mL/min/1.73 m^2^)	−14.8 (−56–16)	−16.5 (−50–24)	0.66
Any recurrence	3 (6.1)	0 (0)	0.13
Local	2 (4.1)	0 (0)	
Metastatic	1 (2.0)	0 (0)	

__^a^GFR, glomerular filtration rate.

## Abbreviations

ASA: American Society of Anesthesiology
GFR: Glomerular filtration rate
LPN: Laparoscopic partial nephrectomy
RFA: Radiofrequency ablation
RFA-RPN: Radiofrequency-assisted robotic clampless partial nephrectomy.

## References

[1] Karumanchi S. A., Merchan J., Sukhatme V. P. (2002). Renal cancer: molecular mechanisms and newer therapeutic options. *Current Opinion in Nephrology and Hypertension*.

[2] Pantuck A. J., Zisman A., Belldegrun A. S. (2001). The changing natural history of renal cell carcinoma. *The Journal of Urology*.

[3] Jewett M. A. S., Zuniga A. (2008). Renal tumor natural history: the rationale and role for active surveillance. *Urologic Clinics of North America*.

[4] Desai M. M., Aron M., Gill I. S. (2005). Laparoscopic partial nephrectomy versus laparoscopic cryoablation for the small renal tumor. *Urology*.

[5] Finley D. S., Beck S., Box G. (2008). Percutaneous and laparoscopic cryoablation of small renal masses. *The Journal of Urology*.

[6] Carraway W. A., Raman J. D., Cadeddu J. A. (2009). Current status of renal radiofrequency ablation. *Current Opinion in Urology*.

[7] Nadler R. B., Perry K. T., Smith N. D. (2009). Hybrid laparoscopic and robotic ultrasound-guided radiofrequency ablation-assisted clampless partial nephrectomy. *Urology*.

[8] Wu S. D., Viprakasit D. P., Cashy J., Smith N. D., Perry K. T., Nadler R. B. (2010). Radiofrequency ablation-assisted robotic laparoscopic partial nephrectomy without renal hilar vessel clamping versus laparoscopic partial nephrectomy: A Comparison of Perioperative Outcomes. *Journal of Endourology*.

[9] Jacomides L., Ogan K., Watumull L., Cadeddu J. A. (2003). Laparoscopic application of radio frequency energy enables in situ renal tumor ablation and partial nephrectomy. *The Journal of Urology*.

[10] Kutikov A., Uzzo R. G. (2009). The R.E.N.A.L. nephrometry score: a comprehensive standardized system for quantitating renal tumor size, location and depth. *Journal of Urology*.

[11] Dindo D., Demartines N., Clavien P.-A. (2004). Classification of surgical complications: a new proposal with evaluation in a cohort of 6336 patients and results of a survey. *Annals of Surgery*.

[12] Gettman M. T., Bishoff J. T., Su L. M. (2001). Hemostatic laparoscopic partial nephrectomy: initial experience with the radiofrequency coagulation-assisted technique. *Urology*.

[13] Corwin T. S., Cadeddu J. A. (2001). Radio frequency coagulation to facilitate laparoscopic partial nephrectomy. *Journal of Urology*.

[14] Sundaram C. P., Rehman J., Venkatesh R. (2003). Hemostatic laparoscopic partial nephrectomy assisted by a water-cooled, high-density, monopolar device without renal vascular control. *Urology*.

[15] Urena R., Mendez F., Woods M., Thomas R., Davis R. (2004). Laparoscopic partial nephrectomy of solid renal masses without hilar clamping using a monopolar radio frequency device. *The Journal of Urology*.

[16] Stern J. A., Simon S. D., Ferrigni R. G., Andrews P. E. (2004). TissueLink device for laparoscopic nephron-sparing surgery. *Journal of Endourology*.

[17] Herrell S. D., Levin B. M. (2005). Laparoscopic partial nephrectomy: use of the tissuelink™ hemostatic dissection device. *Journal of Endourology*.

[18] Oefelein M. G. (2006). Delayed presentation of urinoma after radiofrequency ablation-assisted laparoscopic partial nephrectomy. *Journal of Endourology*.

[19] Coleman J., Singh A., Pinto P. (2007). Radiofrequency-assisted laparoscopic partial nephrectomy: clinical and histologic results. *Journal of Endourology*.

[20] Zeltser I. S., Moonat S., Park S., Anderson J. K., Cadeddu J. A. (2008). Intermediate-term prospective results of radiofrequency-assisted laparoscopic partial nephrectomy: a non-ischaemic coagulative technique. *BJU International*.

[21] Sprunger J., Herrell S. D. (2005). Partial laparoscopic nephrectomy using monopolar saline-coupled radiofrequency device: animal model and tissue effect characterization. *Journal of Endourology*.

[22] Pareek G., Wilkinson E. R., Schutt D. (2005). Haemostatic partial nephrectomy using bipolar radiofrequency ablation. *BJU International*.

[23] Louie M. K., Deane L. A., Kaplan A. G. (2011). Laparoscopic partial nephrectomy: six degrees of haemostasis. *BJU International*.

[24] Syvanthong C., Wile G. E., Zagoria R. J. (2007). Effect of radiofrequency ablation of renal tumors on renal function in patients with a solitary kidney. *American Journal of Roentgenology*.

[25] Gill I. S., Colombo J. R., Moinzadeh A. (2006). Laparoscopic partial nephrectomy in solitary kidney. *Journal of Urology*.

